# Integrative analysis for finding genes and networks involved in diabetes and other complex diseases

**DOI:** 10.1186/gb-2007-8-11-r253

**Published:** 2007-11-28

**Authors:** Regine Bergholdt, Zenia M Størling, Kasper Lage, E Olof Karlberg, Páll Í Ólason, Mogens Aalund, Jørn Nerup, Søren Brunak, Christopher T Workman, Flemming Pociot

**Affiliations:** 1 Steno Diabetes Center, Niels Steensensvej 2, DK-2820 Gentofte, Denmark; 2 Center for Biological Sequence Analysis, Technical University of Denmark, DK-2800 Lyngby, Denmark; 3 Neurotech A/S, DK-2100 Copenhagen, Denmark; 4 Institute for Clinical Science, University of Lund, SE-221 00 Lund, Sweden

## Abstract

We have developed an integrative analysis method combining genetic interactions, identified using type 1 diabetes genome scan data, and a high-confidence human protein interaction network. Resulting networks were ranked by the significance of the enrichment of proteins from interacting regions. We identified a number of new protein network modules and novel candidate genes/proteins for type 1 diabetes. We propose this type of integrative analysis as a general method for the elucidation of genes and networks involved in diabetes and other complex diseases.

## Abstract 2

An integrative analysis combining genetic interactions and protein interactions can be used to identify candidate genes/proteins for type 1 diabetes and other complex diseases.

## Background

Complex traits like type 1 diabetes (T1D) are generally believed to be under the influence of multiple genes interacting with each other to confer disease susceptibility and/or protection. Identification of susceptibility genes in complex genetic diseases, however, poses many challenging problems. The contribution from single genes is often limited and genetic studies generally do not offer clues about the functional context of a gene associated with a complex disorder. A recent report demonstrated the feasibility of constructing functional human gene networks (using, for example, expression and Gene Ontology (GO) data [1]), and using these in prioritizing positional candidate genes from non-interacting susceptibility loci for various heritable disorders [2]. It was shown that the obvious candidate genes were not always involved, and that taking an unbiased approach in assessing candidate genes using functional networks may result in new, non-obvious hypotheses that are statistically significant.

One of the strongest indications of functional association is the presence of a physical interaction between proteins [3] and several reports have shown that proteins involved in the same phenotype are likely to be part of the same functional module (that is, protein sub-network) [4-6]. With this in mind, it seems reasonable to expect that, in many cases, components contributing to the same complex diseases will be members of the same functional modules, especially if the disease is associated with multiple genetic loci that show statistical indication for epistasis. This indicates that in the case of complex disorders a feasible strategy would be to search for groups of interacting proteins that together lead to significant association with the disease in question. However, a strategy searching for loci showing genetic interaction (epistasis) integrated with a search for protein networks spanning the epistatic regions and subsequent significance ranking of these networks has, to our knowledge, never been pursued for any complex disorder.

Presumably, this is because a number of problems are associated with such a strategy. First, traditionally genetic linkage analysis is performed by searching for the marginal effect of a single putative trait locus, whereas methods for searching for multiple trait loci simultaneously are limited [7-11], and in T1D statistical indication for epistasis has been shown only for a few candidate loci [10,12,13]. Secondly, an insufficient amount of human protein interaction data has precluded systematic analyses of protein networks enriched for proteins originating from interacting genomic regions. Moreover, no single database houses all human protein interaction data, and the data are generally noisy, containing many false positive interactions [4]. Thirdly, no standard statistical method for measuring the significance of protein networks, based on the enrichment of proteins from genetically interacting regions, has yet been reported.

**Figure 1:**
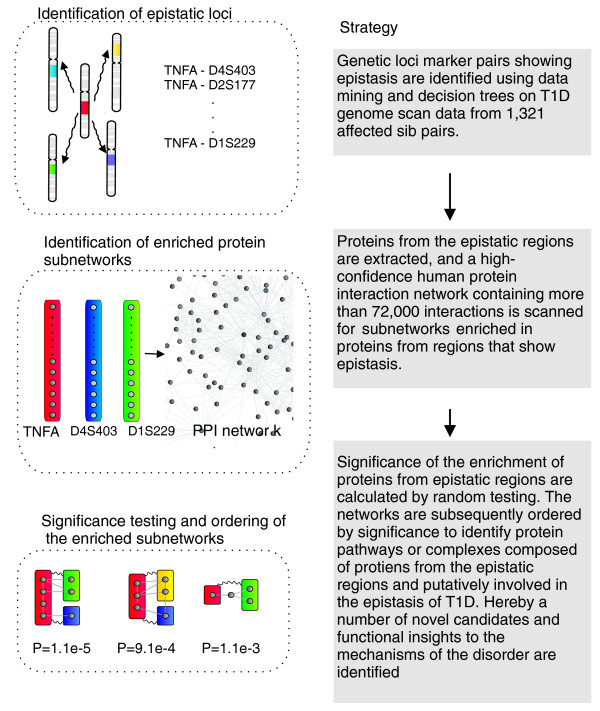
The strategy used for the current study.

We addressed these issues through a number of approaches. First, data mining/decision trees were used to identify genetic markers or combinations of markers of predictive value for T1D. This approach is well suited to handle the complexity of genetic data, and has been proven to be able to precisely identify risk loci associated with T1D, as well as interacting genetic regions [14-18]. In the present study we have tested whether identical-by-descent (IBD) sharing data [19-21], instead of exact allele-calling genotypes as previously used [18], could be used to identify risk loci. The data analyzed were from the published T1D genome scans [22,23] available through the Type 1 Diabetes Genetics Consortium (T1DGC) [24]. We have recently constructed a high-confidence human protein interaction network by extensive data integration, including conservative incorporation of data from model organisms, followed by rigorous quality scoring of the protein interactions [4]. This network was searched for protein networks enriched in proteins from the interacting genetic regions demonstrated. Subsequently, we developed a new statistical method for evaluating the significance of this enrichment, which enabled us to rank all identified networks. The strategy used is outlined in Figure 1.

Several significant networks were identified. Some of the candidates in these networks were known HLA (human leukocyte antigen) region (chromosome 6p21) genes, including the recently identified T1D associated candidate gene *ITPR3*, which was centrally located in one of the top scoring networks. However, some significant networks contained protein components that have never been associated with T1D. Since all candidates identified in the present work were put in a functional context with other members of a network (guilt-by-association), the networks immediately offer clues on the functional role of the candidates and other proteins in relation to T1D. Our observations support that genetic interactions are important in T1D susceptibility, and that an integration of genetic and physical interactions is an interesting new approach for analyzing complex disorders.

## Results

### Marginal markers

In the total data set of 1,321 affected sibling pair families from the UK, the US and Scandinavia, data mining/decision tree analyses identified major T1D predictive signals (marginal markers; Table 1) corresponding to T1D linkage signals found by classic non-parametric linkage analysis [25]. As the original T1DGC publication [25] included data on 254 additional affected sibling-pair families not part of the present analyses, direct comparison of results is not possible. However, substantial agreement existed between the analyses (Table 1). Ranking of markers is according to their T1D predictive signal determined by Pearson's χ^2 ^statistics and corresponding *P *value. As we evaluated only a limited number of the genotyped markers in the total data set, we endeavored to see if supplementary information could be extracted from more complete subsets of data (UK/US and Scandinavian). As seen in Table 1, the group of markers corresponding to the HLA region shows a much higher predictive signal (by several orders of magnitude) than the rest of the markers. *D6S283 *and *D6S300 *are markers for *IDDM15 *(6q21) [26], which in linkage studies generally require separate analysis to differentiate its effect from MHC [25,26]. Markers for the regions 2q31-q33, 16p12-q11.1, 11p15.5, 16q22-q24 and 10p14-q11 identified by linkage analysis [25] also showed high predictive signals in the current study, either in the total data set or in the data subsets (Table 1). In addition, a few new markers were found to show predictive signals (*P *< 0.05) when evaluated independently of chromosome 6 markers, for example, *D17S798*, *D2S125*, *D9S175*, *D8S261 *and *D4S403*. The *D21S270 *marker was identified in the Scandinavian subset and corresponds to a T1D linkage region on chromosome 21, which we have recently identified and fine mapped [22,27]. In the UK/US data set, the 2q31-q33 region (the *CTLA4 *region) seems of higher predictive value than in the total data set (Table 1). *D4S403 *corresponds to a region previously linked to T1D [22,28] containing the *WFS1 *gene associated with Wolfram syndrome (MIM #222300), which involves T1D [28].

**Table 1:** Marginal markers.

	χ^2 ^(2 d.f.)	P value	Position on chromosome in cM	Confirmed from genome scan (LOD - 1 interval) or other references
**Total data set**				
TNFA	142.0	1.5 × 10^-32^	47	6p21 (46-48 cM) [25]
D6S273	77.0	7.0 × 10^-18^	45	
D6S291	58.2	2.2 × 10^-13^	49.5	
D6S276	34.8	3.4 × 10^-8^	44.4	
D6S260	27.1	8.2 × 10^-7^	29.9	
D6S286	21.4	1.6 × 10^-5^	89.8	
D6S283	18.3	0.0001	109.2	[26]
D6S470	15.2	0.0005	18.2	
D6S300	10.6	0.005	103.5	[26]
D17S798	9.8	0.007	53.4	
D2S152	8.7	0.013	188.1	2q31-33 (177-204 cM) [25]
D2S125	7.0	0.03	260.6	
D9S175	6.3	0.043	70.3	
D8S261	6.1	0.048	37.0	
D4S403	6.1	0.048	25.9	
				
**Selected markers**				
UK/US subset				
D2S389	13.1	0.001	190	2q31-33 (177-204 cM) [25]
D16S769	9.4	0.009	50.6	16p12-q11.1 (26-71 cM) [25]
Th1	9.0	0.011	5.9	11p15.5 (0-14 cM) [25]
D16S289	8.1	0.017	105	16q22-q24 (100-121 cM) [25]
D10S183	6.7	0.035	60.6	10p14-q11 (52-66 cM) [25]
				
SCAND subset				
D21S270	6.4	0.039	38.1	[27]

Markers of predictive value for T1D identified by decision tree analysis on T1D genome scan data from 1321 affected sib pair families. Markers identified in the total data set are ranked according to significance level (P < 0.05). Markers from data subsets are 'selected markers' and were selected on basis of whether they confirm loci from the latest T1D genome scan [25] or other references [26; 27]. D.f. = degrees of freedom.

### Epistasis

The importance of HLA is well established, and we are, by the methods used here, able to evaluate important markers in sibling pairs sharing just one HLA allele. The top scoring marginal marker for the HLA region was the tumor necrosis factor alpha (*TNFA*) micro satellite marker, located centrally in the HLA region. To determine candidates for the next level, we searched for interacting markers with the HLA region, in the subgroups of sibling pairs with *TNFA *IBD status = 1 (*TNFA *= 1) and *TNFA *IBD status = 2 (*TNFA *= 2), respectively. No interactions with *TNFA *= 0 could be generated due to the low number of affected sibling pairs in this group. Specific combinations of markers corresponding to statistically significant genetic interactions in the combined data set are shown in Table 2. The marker combination *TNFA *= 1 - *D11S910 *was shown to be of protective value, since sibling pairs sharing one *TNFA *allele, but two alleles of *D11S910*, were strongly protected against T1D (of 25 sibling pairs with this combination, one was concordant for T1D, 24 were non-T1D). The other combinations of markers detected implied increased susceptibility to T1D. None of the interacting markers from Table 2, except *D4S403*, correspond to previously known regions associated with T1D [29].

**Table 2:** Statistically significant genomic interactions

First level	Second level	Pearson's χ^2 ^(2 d.f.)	*P *value
TNFA = 2	D4S403	9.10	0.011
TNFA = 2	D2S177	7.33	0.026
TNFA = 2	D1S229	6.88	0.032
TNFA = 1	D11S910	11.82	0.0027
TNFA = 1	D13S170	6.84	0.033
TNFA = 1	D16S287	6.63	0.036
D17S798 = 2	D2P25	6.41	0.041
D17S798 = 1	D5S429	7.08	0.029
D17S798 = 1	D1S197	6.94	0.031

Markers corresponding to the first and second level of each significant interaction, as well as Pearson χ^2 ^statistics and corresponding *P *value, are shown. Affected sibling pairs (ASPs) genotyped for the TNFA and D17S798 marker were as follows (non-T1D sibling pairs were simulated to be twice the number of ASPs for each group): TNFA = 2, 520 ASP and 1,040 non-T1D sibling pairs; TNFA = 1, 206 ASP and 412 non-T1D sibling pairs; D17S798 = 2, 136 ASP and 272 non-T1D sibling pairs; D17S798 = 1, 254 ASP and 508 non-T1D sibling pairs.

Genetic interaction analysis was performed for the marginal markers with the highest predictive signals, and was also performed independent of HLA (*TNFA*) IBD sharing status. When evaluating epistasis independent of HLA, we searched specifically for epistasis between the three highest ranking markers, *D17S798*, *D2S152 *and *D2S125*, after chromosome 6 markers were removed. In the combined data set, however, only combinations including the marker on chromosome 17 predicted genetic interaction (that is, *D17S798 *= 1 - *D5S429 *(*P *= 0.029) and *D17S798 *= 1 - *D1S197 *(*P *= 0.031), and between *D17S798 *= 2 - *D2P25 *(*P *= 0.041)). These combinations reached statistical significance, and demonstrated increased susceptibility to T1D (Table 2). Relationships could only be inferred for two markers at a time due to the high number of missing and non-informative values for many markers.

### Protein interaction networks

We searched for protein networks spanning the regions shown to interact genetically (*P *values < 0.05; Table 2). This was performed using a high-confidence human protein interaction network [4]. Input proteins were proteins corresponding to a defined genetic region surrounding the interacting markers included in the different marker combinations. For all markers except *TNFA*, 5 Mb on each side of the marker in question was used as input. This region size was selected since linkage peaks (LOD - 1 intervals) from genome scans that use a similar number of markers often corresponded to regions of this size. For the HLA region, we have exclusively used the classic MHC region (4 Mb) for analysis, due to the well examined nature of this region with a high degree of linkage disequilibrium, as well as the large number of genes clustered in this specific region [30]. The classic MHC region comprises the *TNFA *marker in a central position (positioned at bases 31,643,403-31,643,437 on the physical map of chromosome 6, corresponding to 46.7 cM).

**Figure 2:**
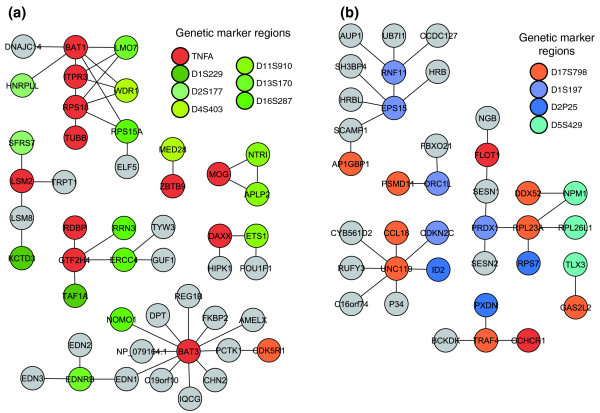
Protein interaction networks for predicted genetic interactions. **(a) ***TNFA-D4S403*, *TNFA-D13S170 *and *TNFA-D2S177 *are represented by one network, whereas *TNFA-D1S229*, *TNFA-D16S287 *and *TNFA-D11S910 *are represented by two or three networks. Color-code: red, genes from *TNFA *region; green and yellow, genes from interacting region; light grey, genes from other chromosomes. **(b) **Protein interaction networks involving *D17S798*. *D17S798-D1S197*, *D17S798-D2P25 *and *D17S798-D5S429 *are represented by four, three and two networks, respectively. Color-code: red, genes from *D17S798*-region; blue/green, genes from interacting region; light grey, genes from other chromosomes.

We were able to identify 22 protein sub-networks that connect proteins from the different regions corresponding to the significant two-marker predicted genetic interactions. The union of these sub-networks resulted in 13 putative functional modules (Figure 2).

### Network significance analysis

**Figure 3:**
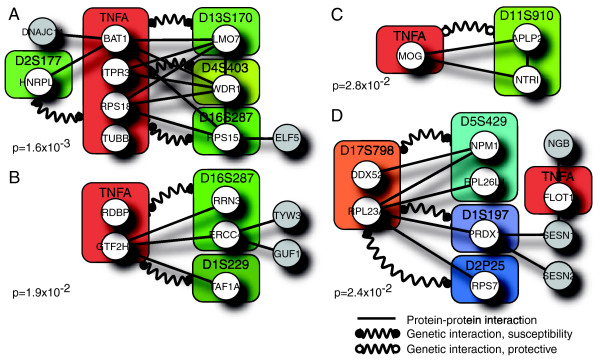
Significant functional modules (modules A-D). Straight lines represent validated protein-protein interactions, curved lines represent demonstrated genetic interactions (black bullets, predictive interactions; white bullets, protective interactions). Circles with gene names represent the gene encoding the protein of the interaction. Boxes are the marker regions shown to be involved in the genetic interactions and in which the genes are located.

The significance of each putative functional module was assessed by comparison to search results for randomly selected genetic regions. This assessment was made for both the results of marker-region pairs (2-interval) and for the resulting merged modules containing genes from two or more intervals (*k*-interval). Four 2-interval modules that included *TNFA*-region genes, two of which were found to be significant, were merged into a single highly significant 5-interval module (Figure 3, module A). This concordance strongly suggests that the four *TNFA*-region genes *TUBB*, *RPS18*, *ITPR3 *and *BAT1 *may be important in explaining the mechanism of the four genetic interactions. From the interacting chromosomal regions, the *WDR1*, *LMO7*, *HNRPLL *and *RPS15A *genes are potential T1D candidate genes. These genes are involved in transcriptional regulation, DNA binding, RNA binding, ion channel activity, ATP synthesis, actin binding and natural killer cell mediated cytotoxicity and cell proliferation. Candidate genes from the four significant functional modules (Figure 3) are listed in Table 3. Other networks with *TNFA *include genes involved in signal transduction, regulation of transcription, protein biosynthesis and folding, histone activity, ubiquitin-protein ligase activity, as well as response to oxidative stress (Table 3), also of potential relevance in T1D pathogenesis.

**Table 3:** Genes corresponding to protein interactions in the four statistically significant functional modules A, B, C and D (in Figure 3)

Gene name	Chromosomal band	Description	GO term
**Module A**			
*DNAJC14*	[12q13.2]	Nuclear protein Hcc-1 (Proliferation associated cytokine-inducible protein CIP29)	Heat shock protein binding, unfolded protein binding
*HNRPLL*	[2p22.1]	Heterogeneous nuclear ribonucleoprotein L-like (Stromal RNA-regulating factor)	Nucleotide binding, RNA binding, mRNA processing
*BAT1*	[6p21.33]	Spliceosome RNA helicase BAT1 (HLA-B associated transcript-1)	Nucleotide binding, nucleic acid binding, ATP-dependent RNA helicase activity, nuclear mRNA splicing, mRNA export from nucleus, ATP biosynthetic process, ion transport
*ITPR3*	[6p21.31]	Inositol 1,4,5-trisphosphate receptor type 3	Ion channel activity, calcium channel activity, calcium ion transport, protein binding, signal transduction
*RPS18*	[6p21.32]	40S ribosomal protein S18 (Ke-3)	RNA binding, structural constituent of ribosome, rRNA binding, translation
*TUBB*	[6p21.33]	Tubulin beta-2 chain	Nucleotide binding, GTPase activity, cell motility, natural killer cell mediated cytotoxicity
*LMO7*	[13q22.2]	LIM domain only protein 7 (LOMP) (F-box only protein 20)	Protein ubiquination, actomyosin structure and biogenesis, protein binding, ion binding
*WDR1*	[4p16.1]	WD repeat domain 1 (WDR1), transcript variant 1	Actin binding, protein binding, sensory perception of sound
*RPS15A*	[16p12.3]	40S ribosomal protein S15a	Protein binding, structural constituent of ribosome, translation
*ELF5*	[11p13]	ETS-related transcription factor Elf-5 (E74-like factor 5)	Transcription factor activity, sequence-specific DNA binding, regulation of transcription, cell proliferation
			
**Module B**			
*RDBP*	[6p21.3]	RD RNA-binding protein, MHC complex gene RD	RNA binding, nucleotide binding, transcription, regulation of transcription
*GTF2H*	[2q14.3]	Basic transcription factor 2 89 kDa subunit, DNA excision repair protein ERCC-3	DNA binding, ATP-dependent DNA helicase activity, transcription-coupled nucleotide-excision repair, regulation of transcription
*RRN3*	[16p13.11]	RNA polymerase I-specific transcription initiation factor	RNA polymerase I transcription factor activity, regulation of transcription
*ERCC4*	[16p13.12]	DNA excision repair protein, DNA repair endonuclease	DNA binding, magnesium ion binding, nucleotide excision repair
*TAF1A*	[1q41]	TATA box binding protein (TBP)-associated factor, RNA polymerase I	DNA binding, RNA polymerase I transcription factor activity, regulation of transcription
*TYW3*	[1p31.1]	tRNA-yW synthesizing protein 3 homolog	None
*GUF1*	[4p13]	GTP-binding protein GUF1 homolog, GTPase of unknown function	Nucleotide binding, translation initiation factor activity, GTPase activity, small GTPase mediated activity
			
**Module C**			
*MOG*	[6p22.1]	Myelin-oligodendrocyte glycoprotein precursor	Synaptic transmission, central nervous system development
*APLP2*	[11q24.3]	Amyloid-like protein 2 precursor (APPH)	DNA binding, protein binding, G-protein coupled receptor protein signaling pathway
*NTRI*	[11q25]	Neurotrimin precursor (hNT)	Protein binding, cell adhesion, neuron recognition
			
**Module D**			
*DDX52*	[17q12]	Probable ATP-dependent RNA helicase DDX52 (DEAD box protein 52)	Nucleotide binding, ATP binding, ATP-dependent helicase activity
*RPL23A*	[17q11.2]	60S ribosomal protein L23a	Nucleotide binding, rRNA binding, translation
*NPM1*	[5q35.1]	Nucleophosmin (NPM) (Nucleolar phosphoprotein B23)	Transcription coactivator activity, RNA binding, intracellular protein transport, anti-apoptosis, response to stress
*RPL26L1*	[5q35.1]	60S ribosomal protein L26-like 1	Structural constituent of ribosome, translation
*PRDX1*	[1p34.1]	Natural killer cell-enhancing factor A, Peroxiredoxin-1	Oxidoreductase activity, peroxiredoxin, cell proliferation
*RPS7*	[2p25.3]	40S ribosomal protein S7	RNA binding, protein binding, translation
*NGB*	[14q24.3]	Neuroglobin	Oxygen transporter activity, metal ion binding
*FLOT1*	[6p21.33]	Flotillin 1, integral membrane component of caveolae	Protein binding
*SESN1*	[6q21]	Sestrin-1 (p53-regulated protein PA26)	Response to DNA damage stimulus, cell cycle arrest, negative regulation of cell proliferation
*SESN2*	[1p35.3]	Sestrin-2, hypoxia induced gene 95 (Hi95)	Cell cycle arrest

Genes corresponding to protein interactions in the four statistically significant functional modules A, B, C and D (in Figure 3). Gene names, chromosomal bands, short descriptions and gene ontology terms (molecular function and biological process) are provided.

A region on chromosome 17 also conferred a high predictive value for T1D and was found to have genetic interactions with three other marker regions. Searches conducted for genes from the three marker pairs (*D17S798-D2P25*, *D17S798-D5S429 *and *D17S798-D1S197*) resulted in six putative functional modules after the initial results were combined (Figure 2). Several of the proteins in these networks are involved in signal transduction, anti-apoptosis, RNA binding regulation of transcription, kinase activity, oxidoreductase activity, DNA and ATP binding as well as oxygen transporter activity (Table 3), making them potentially important in T1D pathogenesis. One of these modules (Figure 3, module D) was found to be significant (*P *< 0.05) and contained protein interactions between members of three genetic interaction marker pairs. GO terms for molecular function and biological process for all candidate genes in significant functional modules are listed in Table 3. These findings shed light on the pathways the candidate genes in these two regions are likely to be involved in, and may help in understanding the possible effect in T1D suggested by this interaction.

## Discussion

Identifying genes in multi-factorial diseases is difficult. Studies in model organisms suggest that epistasis may play an important role in the etiology of multifactorial diseases and complex traits in humans. There is no consensus as to the best strategy for detecting epistatic interactions in humans [31,32]. Several recent studies in humans and animals have identified loci that interact significantly but contribute little or with no effect individually [33-35]. In T1D, attempts to elucidate possible epistasis between classic T1D loci in humans, as well as animal models, have provided only a few examples [10,12,13]. This highlights the need for new methods in detecting and characterizing epistasis, as well as elucidating the presumed underlying biological interactions [31,32]. In the present study we confirmed that the application of data mining methods identified most major signals (marginal markers) found using classic non-parametric linkage analysis [25]. A special feature of the methods used in the current study is that interactions can be generated with marker IBD = 1 and IBD = 2 status. No marker combination with marker IBD = 0 could be generated (due to a low number of affected sibling pairs in this group).

We demonstrated several significant interactions between two different markers predictive for increased susceptibility to T1D and one rule (*TNFA *= 1 - *D11S910*), which predicted protection against T1D. Generation of specific combinations of markers between different chromosomal regions supports that interaction is important in complex diseases like T1D. A number of recent efforts have combined linkage mapping with the identification of co-regulated genes using microarrays to discover trans-acting expression quantitative trait loci [36-39]. While this may be a promising approach also for identifying epistatic susceptibility genes in multifactorial diseases like T1D, data for combined genetic and gene expression studies in T1D are still limited.

In our effort to identify the cellular systems underlying the genetic interactions, we constructed protein sub-networks that spanned the interacting regions to investigate whether the gene products in these regions could be shown to physically interact. The resulting networks were subsequently statistically tested based on the significance of the enrichment of proteins from interacting regions. After merging results for common marker regions (*TNFA *and *D17S798*), it was possible to identify four high-confidence protein interaction sub-networks that were significantly enriched in proteins from regions that interact, thereby supporting all nine epistatic combinations identified. The constructed networks point to specific candidates, and functional relationships between the candidates. Further supporting the importance of the most significant *TNFA *functional module reported here (Figure 2a), a recent paper mapped the *ITPR3 *gene in the HLA region as a new candidate gene for T1D [40], since strong genetic association was demonstrated in two Swedish case-control cohorts.

Additionally, when all chromosome 6 markers were removed, we inferred genetic interactions for regions on chromosomes 1, 2 and 5 interacting with a region on chromosome 17. A single significant functional module resulted after combining results from the three marker-pair searches that included *D17S798*. This functional module implicated a physical interaction between one protein from all three associated regions with a protein encoded by the *RPL23A *gene.

We hypothesize that the significant functional modules elucidated in this current study represent critical steps in pathways of relevance in T1D pathogenesis. The identification of known T1D associated genes supports the value of this method in searching for yet unidentified genetic and functional interactions involved in the pathogenetic processes leading to complex genetic diseases.

Most of the genes encoding proteins of the functional module networks have GO terms [1] (Table 3). However, most GO terms for molecular function and biological processes relate to each other in a simple manner and the current study supports that regulation of transcription and translation, signal transduction, ATP binding, and DNA and RNA binding are of relevance for beta-cell destruction in T1D pathogenesis (Table 3). The functional implications for the protein-protein interactions predicted strengthens the findings and highlights specific genes as candidates for further analysis. With 30% or more of human genes lacking functional annotation, existing protein interaction databases and maps are still far from being complete. Although many of the protein interactions in databases have not been rigorously tested and validated, in this work we applied very strict thresholds, including only protein interactions that were supported by various independent data sources. The functional modules presented in this study also allow for the prediction of specific candidate genes and proteins that may explain the nature of the observed genetic interactions.

## Conclusion

The data presented in the current study comprise, to our knowledge, the most extensive genetic epistasis analysis in a multifactorial disease (T1D) supported by protein interaction networks. It is the first integration of genetic interactions with a systematic search for physical protein interaction networks significantly enriched in proteins from the interacting regions. The results point to specific positional candidates and cellular systems that may underlie disease susceptibility. We believe the genetic interactions produced here and the specific candidates and molecular systems highlighted by our protein network analysis will lead to new insight into the molecular pathology of T1D. Furthermore, we propose our integrative analysis as a general method for the analysis of genes and systems involved in various complex disorders.

## Materials and methods

### Genome scan data

The data set was generated by T1DGC as part of the combined analysis of the existing T1D genome scans [22,23,25]. In this process all genotyping data were intra-familially recoded, when possible, to show IBD status rather than exact allele calls. The Scandinavian data set comprised 392 families (411 affected sibling pairs) that were genotyped for 335 microsatellite markers. The combined UK/US data set included 763 families (910 affected sibling pairs) and genotyping of 1,283 markers. In order to analyze markers only genotyped in all data sets the number of markers was reduced to 298. Thus, the total data set used in the analysis comprised 1,321 affected sibling pairs with genotyping data on 298 markers.

### Data simulation for non-affected sibling pairs

As the total data set included only a few unaffected sibling pairs and the analytical methods applied in the present study take advantage of information from non-diseased subjects [18], we simulated data for non-affected sibling pairs [14-17,41]. A data matrix for unaffected sibling pairs was generated from the data matrix representing the affected sibling pairs. For each marker the number of missing values from the affected was maintained for unaffected sibling pairs. The rest of the matrix for unaffected sibling pairs was completed with values reflecting normal IBD 0, 1 and 2 frequencies, that is, 0.25, 0.5 and 0.25. No correction was made in the simulation for the actual frequency of homozygous parents. The number of unaffected sibling pairs (simulated) was two times the number of affected sibling pairs. The final data matrix then contained 1,311 affected sibling pairs and 2,622 non-affected sibling pairs.

### Analyses: marginal markers and interactions

Identification of marginal markers and evaluation of interaction between markers were done as detailed previously [18], with minor modifications. Briefly, data mining algorithms and decision trees were used to predict the most informative markers. We have used the concept of marginal markers and the interactive tree model in SAS Enterprise Miner (SAS Institute Inc., Cary, NC, USA) to calculate all marginal markers using Pearson's χ^2 ^statistics and corresponding *P *value. The tree algorithm determines marginal markers as the roots (the highest level of the trees), as described previously [18]. The list of marginal markers identified by this method is produced by Pearson's χ^2 ^statistics and corresponding *P *value.

When searching for interactions between a marginal marker and markers on different chromosomes, we also used Pearson's χ^2 ^statistics. Data sets were created including sibling pairs with *TNFA *IBD status = 1 (*TNFA *= 1), *TNFA *IBD status = 2 (*TNFA *= 2), *D17S798 *IBD status = 1 (*D17S798 *= 1) and *D17S798 *IBD status = 2 (*D17S798 *= 2) to search for interactions between these, the highest ranked, marginal markers and other markers. Pearson's χ^2 ^statistics was then used to search for association between T1D and a marker in these individual data sets. Searching for interactions between markers on the same chromosome was not performed, because the random methods used here do not allow for linkage disequilibrium of adjacent markers on a chromosome.

### Human protein interaction networks

A human protein interaction network was generated [4]. Briefly, protein interaction data were obtained from the databases BIND [42,43], MINT [44], IntAct [45], KEGG annotated protein-protein interactions (PPrel), KEGG Enzymes involved in neighboring steps (ECrel) [46] and Reactome proteins involved in the same complex, indirect complex or same or indirect reaction [47]. All human data were pooled, and to increase information interolog data (protein interactions among orthologous protein pairs in different organisms) from 17 eukaryotic organisms were also included to obtain protein-protein interaction networks [4]. We devised and thoroughly tested a global confidence score for all interactions in the network. This confidence score is constructed to take into account factors like topology of the interaction network surrounding the interaction, number of publications the interaction had been detected in, that interactions are more reliable, if they have been reproduced in more than one independent interaction experiment, and, furthermore, the experimental set-up (large- or small-scale study). Interactions from large-scale experiments generally contain more false positives than interactions from small-scale experiments [48]. Furthermore, the reliability of this score was confirmed by fitting a calibration curve of the score against overlap with a high-confidence set of about 35,000 human interactions, demonstrating that the score was a reliable measure of interaction confidence [4]. Networks were constructed from proteins in defined intervals (corresponding to the respective rules) and their first order interaction partners using interolog data in a manner similar to that described by Lehner and Fraser [49]. Proteins known to interact in other species were mapped to their human orthologs using the Inparanoid database [50,51]. In the resulting networks, each node represents all proteins encoded by a single human gene and their orthologs in other species. An edge between two nodes indicates one or more interactions between any of the proteins represented by the node. The protein interaction confidence score was implemented to use only interaction data above the interaction threshold separating 'high' from 'low' confidence interaction data. This threshold was found by using a genetic algorithm on the interaction network to obtain the optimal threshold for signal to noise ratio [4].

To further reduce noise in the networks we also devised a network score, implemented to retrieve sub-networks enriched in proteins from the selected regions that interact directly or through significant linker proteins (that is, proteins that connect proteins from the selected regions, but are not in any of the selected regions themselves). The network score reflects the amount of interaction partners allowed for each linker protein for it to be included in relation to the number of interaction partners from the selected regions. The score is calculated for every protein and is the result of 'number of interactions with input proteins' divided by 'total interactions' for each protein, making networks consisting of proteins with many interactions less important and reducing noise from highly interacting proteins from unselected regions in the genome. A very stringent threshold-score of 0.5 was used.

Positional genes and their corresponding proteins were obtained from the University of California Santa Cruz (UCSC) genome browser using 'Genes and Gene Prediction Tracks' [52] and 'Ensembl Genes' from the table browser [53]. For two marker rules, proteins encoded by genes from 5 Mb on each site of the respective markers were used as input proteins. For the *TNFA *marker, proteins encoded by genes from an interval corresponding to the classic MHC region (position 29.26-33.90 Mb on chromosome 6) [29] were used.

For each protein belonging to an interval of interest, a query was made in the constructed human interaction network. Only interactions above the high-confidence threshold were maintained. Cytoscape version 2.3.1 was used to visualize the resulting networks [54]. Genes were classified according to GO terms [1].

### Statistical assessment of functional modules

In an effort to determine the significance of the putative functional modules, we empirically estimated the probability of observing as many or more marker interval genes (*n*_*i *_and *n*_*j *_for interval *i *and *j*) in modules of size *N *or smaller in our protein interaction network *G*, that is:

*P*(*x*_*i *_≥ *n*_*i*_, *x*_*j *_≥ *n*_*j*_, *X *≤ *N*|*G*).

This probability was estimated for each module with *n*_*i *_> 0 and *n*_*j *_> 0 found for queries based on genes from one of the nine 2-interval genetic interactions. Estimates were derived from the size and number of modules discovered from 100,000 random queries. Random queries were constructed from genes selected from random interval pairs with the same number of genes as in the two genetically interacting marker intervals. Random intervals were defined from consecutive genes on a chromosome. As each query generates a varying number of modules (connected components), the probability estimates were calculated from the frequency of queries that result in one or more connected components containing *x*_*i *_≥ *n*_*i *_and *x*_*j *_≥ *n*_*j *_genes from random interval *i *and *j*, respectively, with total number of *X *≤ *N *genes.

After merging all connected components from the nine different 2-interval queries, six modules were found to contain genes from three or more intervals. For each of these *k*-interval modules, a new set of 100,000 random queries were performed with *k *random intervals of the same sizes as the actual intervals. Probabilities were estimated in the same way as before but now based on gene counts from *k *intervals where *k *ranged from 3 to 5.

## Abbreviations

GO, Gene Ontology; HLA, human leukocyte antigen; IBD, identical by descent; MHC, major histocompatibility complex; T1D, type 1 diabetes; T1DGC, Type 1 Diabetes Genetics Consortium; TNF, tumor necrosis factor.

## Authors' contributions

Basic idea and protocol formation: RB and FP. Data mining and decision tree analyses: MA, RB and FP. Analysis and interpretation of gene-gene interaction data: RB, MA and FP. Development of integrative system for protein-protein interactions: ZMS, KL, EOK, PÍÓ and SB. Construction of protein-protein interaction networks: ZMS, KL, CTW, EOK, PÍÓ and SB. Development of statistical method for ranking of networks: CTW. Interpretation of protein-protein interaction network data: RB, ZMS, KL, CTW and FP. Manuscript preparation: RB with contributions from all authors, mainly from KL, CTW, ZMS, JN and FP.
